# Reports in Brief of Four Cases in Surgery, and One in Obstetrics in Which Chloroform Was Beneficially Used

**Published:** 1848-07

**Authors:** M. Z. Kreider

**Affiliations:** Lancaster, Ohio.


### ARTICLE III.

*Reports in brief of four cases in Surgery, and one case in Obstetrics in which Chloroform was beneficially used.* By M. Z. Kreider, M. D., of Lancaster, Ohio.

Case 1. Amputation of the right mamma for cancer in a married female, aged about thirty years.

The irritation produced by the chloroform about the mouth and nose in this case led me at once to doubt its purity. I afterwards found my suspicions to be well founded, that it did contain a small quantity of absolute alcohol. In my experiments I found that by dropping a small quantity into distilled water in a thin glass vial, it became opaque—in other words the globule of chloroform seemed to become coated with a *milky pellicle*, which after a time disappeared. With pure chloroform this appearance never obtained.

These experiments were made about the middle of February, and before the publication of a like test in France.

The agent though not pure in this case, so far succeeded as to produce insensibility to pain during the operation.

Case 2. An old lady aged 56, suffering from false aneurism produced by wound in bleeding from the arm. Chloroform was administered in this case, producing the full anesthetic condition—the humeral artery was ligated and the patient speedily recovered.

Case 3. A boy aged four years suffering from paraphimosis of six days continuance. The parts were much inflamed, swollen and painful. Administered chloroform which produced insensibility to pain and made the necessary incision to relieve the *glans*. Patient recovered without an untoward symptom.

Case 4. A lady aged 30 years was suddenly attacked with a violent pain in the palm of the hand. The hand became turned, and the pain was so excruciating as to immediately threaten loss of life.

Chloroform was administered, an incision made down to the bone at the point where pain was first felt. The most marked and speedy relief followed and the lady recovered rapidly. The invasion in this case was undoubtedly erysipelatous.

Case 5. A lady in labor five days. Presentation of the left hip. Parts exceedingly swollen, dry and tender—abdomen also very tender to the touch, the consequence of long and severe uterine pain. Chloroform administered—feet brought down, and child delivered alive. Patient speedily recovered.

